# Surgical Challenge in the Management of Spontaneous, Bilateral, Nontraumatic, Neglected Femoral Neck Fractures in a Patient With End-Stage Renal Disease: A Case Report and Literature Review

**DOI:** 10.7759/cureus.38633

**Published:** 2023-05-06

**Authors:** Dareen Rednah, Omer S Brinji, Mishary Aldakhil, Elaf Alshareef, Mohammed Alshehri

**Affiliations:** 1 Orthopaedic Surgery, My Clinic, Jeddah, SAU; 2 Orthopaedic Surgery, King Abdulaziz Medical City, Jeddah, SAU; 3 Orthopaedic Surgery, King Abdulaziz Medical City, Riyadh, SAU; 4 Intensive Care Unit, King Abdulaziz Medical City, Jeddah, SAU; 5 Orthopaedic Surgery, King Saud Bin Abdulaziz University for Health Sciences, Jeddah, SAU

**Keywords:** end-stage renal disease (esrd), total hip arthroplasty, osteoporosis, renal osteodystrophy, bilateral, femoral neck fracture

## Abstract

Renal osteodystrophy is a spectrum of diseases that affect several organ systems including the musculoskeletal system by decreasing bone density which increases the risk of fractures. Fractures around the femoral neck are usually traumatic and unilateral and, rarely, bilateral and atraumatic. In this report, we present the case of a 37-year-old female patient with a known history of chronic kidney disease who sustained an atraumatic bilateral neck of femur fracture with late presentation. In addition, we present a review of neglected femoral neck fracture management in a young patient with renal disease and osteoporosis.

## Introduction

Renal osteodystrophy is associated with several orthopedic manifestations. Some of these include osteomalacia, avascular necrosis, tendinitis, and pathologic fractures, among others. Spontaneous pathological femoral neck fractures that occur as a result of uremic osteodystrophy caused by chronic renal failure have been frequently reported in the literature [1]. However, there are limited reports of bilateral femoral neck fractures in such patients. Seizure disorders and other factors including osteoporosis and trivial or electrical trauma can lead to such fractures as well.

In this article, we present the case of a 37-year-old female with a known seizure disorder and chronic kidney disease. She had a history of simple trauma two months before presentation, leading to bilateral groin pain. On investigations, she was found to have bilateral femoral neck fractures. She underwent bilateral total hip replacement in two stages. The pathophysiology, treatment of the general condition, specific arthroplasty management options, and complications of bilateral femoral neck fractures in patients with end-stage renal disease are discussed here.

## Case presentation

The patient was a 37-year-old female with a history of seizure disorder secondary to electrolyte imbalance. The electrolyte imbalance was attributed to chronic kidney disease for which she refused to undergo dialysis during the early stages. She presented to the emergency room with a history of a simple fall on her side two months prior, which resulted in pain over both hip joints. There was no history of head injury or loss of consciousness. She sought medical advice after the fall, but the results of imaging were not significant at the time as no fractures were seen on imaging. She was discharged from the hospital. Later, her pain became aggravated, and she was bedridden and unable to carry her body weight. Past medical history revealed a urinary tract infection, which was treated with intravenous antibiotics. Although she was diagnosed with end-stage renal disease, she refused to undergo dialysis, which resulted in electrolyte imbalance causing multiple seizure attacks. She was initiated on phenytoin therapy before the fall.

Additionally, she had undergone multiple blood transfusions with episodes of transfusion-related reactions after the transfusion. Her regular medications included enoxaparin, amlodipine, phenytoin, alfacalcidol, omeprazole, folic acid, ferrous sulfate, calcium, and vitamin D supplements. On examination, her vital parameters were stable with a temperature of 36°C, respiratory rate of 20 breaths per minute, heart rate of 113 beats per minute, blood pressure of 108/53 mmHg, and oxygen saturation of 99-100% in room air. She was bedridden, alert, conscious, and oriented. Her musculoskeletal examination showed no open wounds around both hips and no swelling. There was atrophy of the calf muscles and bilateral hip tenderness, more on the right side. The range of motion of both hips was limited due to pain and muscle weakness. Knee and ankle examinations were unremarkable.

Laboratory investigations showed low hemoglobin, sodium, calcium, and phosphate; high parathyroid hormone (PTH), alkaline phosphatase, and creatinine; and normal vitamin D and blood urea nitrogen (BUN) (Table 1).

**Table 1:** Laboratory values upon admission.

Test	Value	Reference range	Comment
Hemoglobin	7.0	12.1–15.1 mg/dL	Low
Sodium	120	136–145 mg/dL	Low
Calcium	2.18	8.6–10.3 mg/dL	Low
Phosphate	2.56	2.8–4.5 mg/dL	Low
Vitamin D	57.2	30–50 ng/mL (optimal), 50–70 ng/mL (upper normal)	Normal
Parathyroid hormone	157.70	10–55 pg/dL	High
Alkaline phosphatase	555	44–147 IU/L	High
Blood urea nitrogen	13.3	10–22 mg/dL	Normal
Creatinine	3.57	0.74–1.35 mg/dL	High

**Figure 1:**
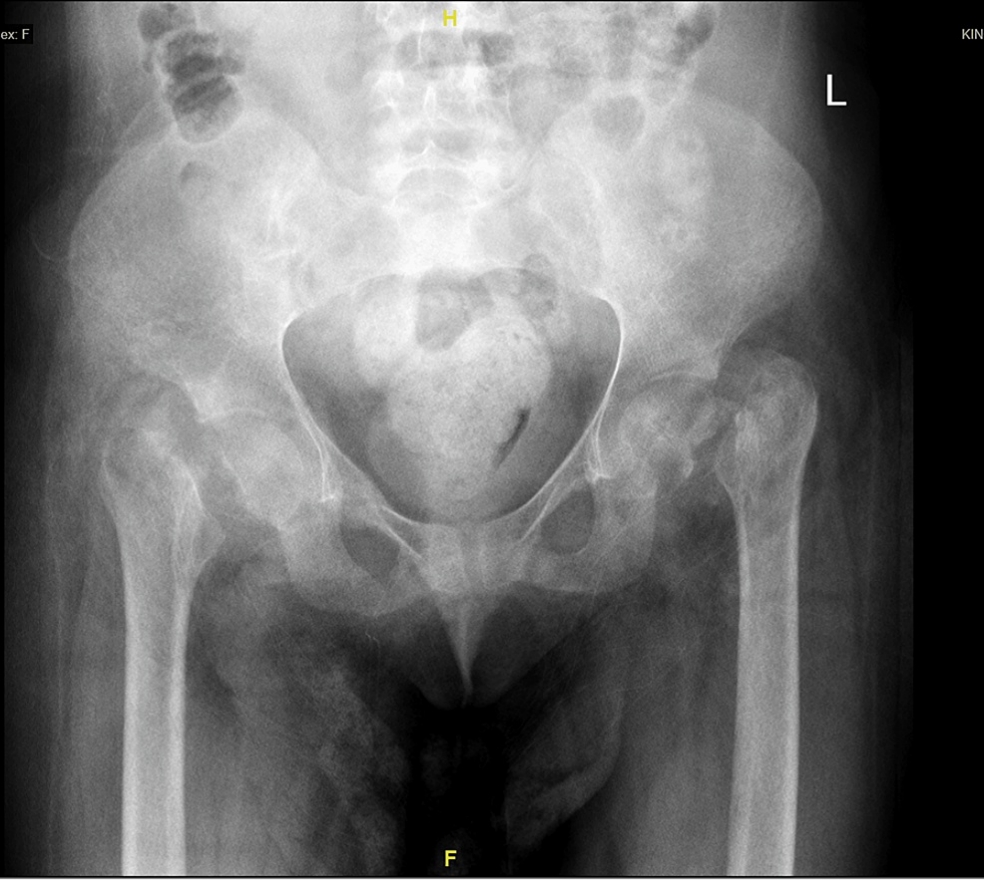
Radiograph of the pelvis on presentation to the emergency room two months after the injury showing bilateral femoral neck fractures.

**Figure 2:**
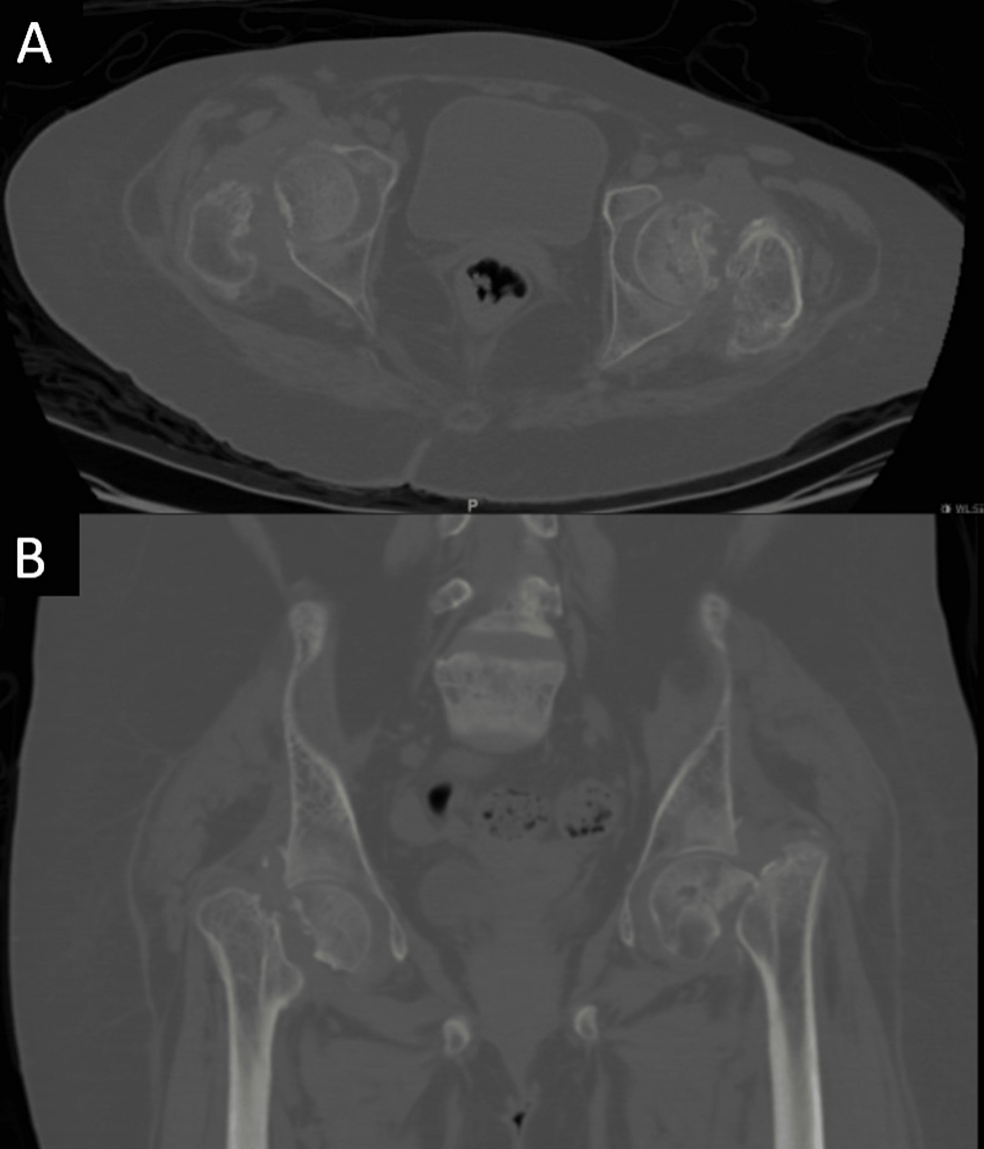
Computerized tomography cuts of both hips showing decreased joint spaces with the absorption of both femoral necks. A: Axial view. B: Coronal reconstruction.

Plain radiographs of both hips showed bilateral, old, subcapital femoral neck fractures with proximal migration of the femur, which was more pronounced in the left hip. A computerized tomography (CT) scan was performed to assess both acetabula and hip joints, which showed narrowing of both joint lines with sclerosis, indicating bilateral hip arthritis (Figures 1, 2).

The patient’s general condition preoperatively was optimized in collaboration with other multidisciplinary services, including nephrology, general internal medicine, neurology, dermatology, and endocrinology.

The patient also underwent treatment for hair lice twice with permethrin. The hair lice did not respond to malathion shampoo, and the surgery was postponed for one week. Finally, the dermatologist advised shaving the hair to eradicate lice. Infectious disease services were consulted as well as she developed a urinary tract infection.

**Figure 3:**
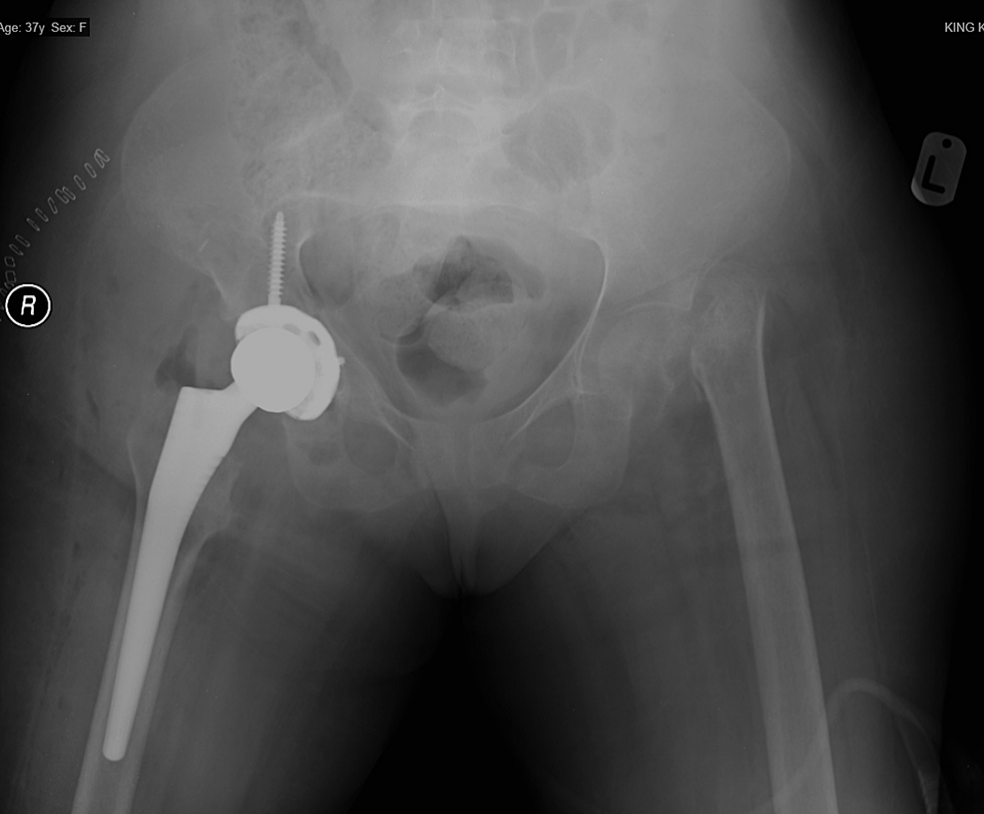
After total arthroplasty of the right hip in 2017.

**Figure 4:**
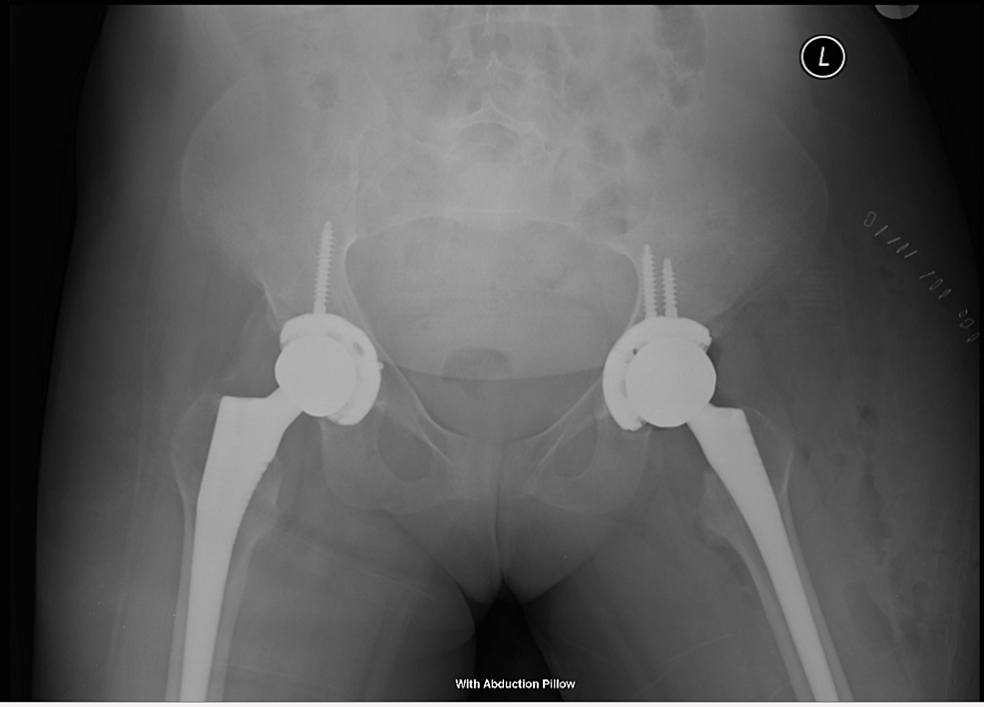
X-ray of the pelvis in 2018 after the second surgery.

**Figure 5:**
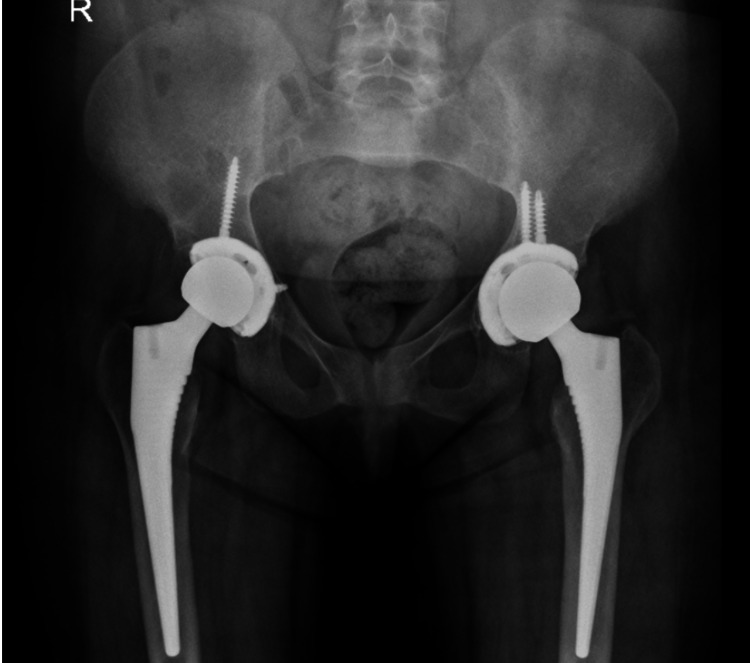
Radiograph of the pelvis seven months after the second total hip arthroplasty with no unusual features or major complications evident.

The initial plan was to perform a single-stage bilateral total hip arthroplasty (THA); however, intraoperatively, the bone quality was found to be poor, and a two-stage THA was opted for to monitor the outcome of the first surgery and avoid complications related to simultaneous bilateral THA. Right-sided uncemented total hip replacement was performed first in 2017. After the surgery, the patient was recovering well with no complications. Dual-energy X-ray absorptiometry scan showed severe osteoporosis; the T-score was -4, and she was started on calcium carbonate and alfacalcidol. Hence, she was discharged and sent for a post-surgery rehabilitation program. Three months later in 2018, she was re-admitted to undergo elective left-sided uncemented total hip replacement. She tolerated the procedure well with no complications and was compliant with the rehabilitation program post-surgically. One month after the surgery, she was seen walking into the clinic with no new complaints and was satisfied with the results. During the two years of follow-up, she remained asymptomatic and was completely mobile without the need for any assistance. Her X-rays were satisfactory with no signs of loosening. The patient continued her follow-up for osteoporosis, was compliant with medications, and was added to the renal transplantation list (Figures 3-5).

## Discussion

Bilateral femoral neck fractures are rare musculoskeletal injuries and are extremely uncommon in cases with no history of trauma. Osteoporosis, bisphosphonate-induced fractures, chronic *Brucella *infections, hypervitaminosis A, cancer, periprosthetic-induced fractures, renal failure-induced osteodystrophy, neuromuscular diseases such as cerebral palsy, and metabolic disease are risk factors for spontaneous fractures [1,2]. Most studies reported that the incidence of fractures progressively increased by 15.0, 20.5, 24.2, 31.2, and 46.3 per 1,000 person-years for chronic kidney disease stages 1, 2, 3a, 3b, and 4, respectively [3]. The risk of hip fractures as reported by the United States Renal Data System was found to be four times higher in those with renal failure and undergoing hemodialysis than in normal subjects [4]. The need for hemodialysis increases the risk of fragility fractures in patients with end-stage renal disease. However, other factors that increase the risk of fractures in end-stage renal disease patients are similar to those for osteoporosis [5]. The use of antiepileptic medications is associated with a 90% increase in the risk of hip fractures due to low bone mineral density (BMD) induced by the medications [6]. In addition, other risk factors include low BMD, low vitamin D (<30 ng/mL), high serum phosphate, and high parathyroid hormone [3]. The five-year mortality rate of such patients, as reported by Klein et al., was 38% [7].

Preoperative optimization and treatment of any source of infection are important in a patient scheduled to undergo arthroplasty to avoid complications such as periprosthetic infection. Hair lice (*Pediculus capitis*) have been associated with bacterial infections due to excessive excoriation in neurosurgery procedures, leading to wound dehiscence [8]. However, to our knowledge, hair lice-related infections related to orthopedic surgeries have not been reported, and there is no clear recommendation about the cancellation of surgery due to hair lice infestation. Surgical management of femoral neck fractures is the preferred option to avoid complications of prolonged non-mobilization such as bed sores and deep vein thrombosis.

However, multiple methods of fixation have been reported in end-stage renal disease patients with femoral neck fractures. Surgical options include osteosynthesis fixation, hemiarthroplasty, or THA. The outcomes of these procedures in end-stage renal disease patients are different compared to those in the normal population. End-stage renal disease patients undergoing osteosynthesis for femoral neck fractures are at an increased risk for non-union and the need for a revision arthroplasty surgery compared to the normal population [9]. End-stage renal disease patients who underwent hemiarthroplasty for femoral neck fractures experienced loosening of the stem regardless of cementing, which was related to poor bone quality in these patients [10,11]. Another option is THA, which is reported to have good functional outcomes and patient satisfaction. The rates of aseptic loosening, dislocation, and revision were reported to be higher in cases of cemented THA, possibly due to inadequate cement-bone interference as a result of poor bone quality in end-stage renal disease patients. In these patients, uncemented THA was found to be superior, with significantly fewer complications and good pain control [12-14]. One-stage simultaneous THA was found to be more economical but was associated with a higher rate of post-surgical complications such as pulmonary embolism (PE), pulmonary infections, and myocardial infarction (MI). Two-stage THA in bilateral femoral neck fracture was found to be safer, especially in patients with comorbidities such as renal failure and old age. PE was not statistically different between the two groups [15].

Detailed complications of each method have been reported. Karaeminogullari et al. reported high rates of non-union in patients with end-stage renal disease who underwent osteosynthesis fixation with screws, almost three years post-surgery with a mortality rate of about 50%, while complications were lower in the arthroplasty group [16]. Kalra et al. reported that rates of non-union and avascular necrosis increased with fixation. Moreover, they found that Austin Moore prosthesis in renal failure patients increased the rate of revision surgeries [17]. In two years of follow-up, Puvanesarajah et al. found that the rate of infection increased in both fixation and hemiarthroplasty groups. The rate of transfusion and mortality increased in both groups in the first- and second-year post-surgery [18]. Bleeding was another complication associated with hemiarthroplasty in end-stage renal disease patients, which necessitated additional surgery for the evacuation of the hematoma. This was due to coagulopathy associated with renal failure and the use of low-molecular-weight heparin as prophylactic post-surgery. MI, PE, infections not responding to debridement alone and necessitating removal of the implant, pneumonia, urinary tract infection, and sepsis were other complications associated with hemiarthroplasty in end-stage renal disease patients [10,19]. Stem migration in cemented hemiarthroplasty was reported in most cases in the first three years post-surgery in patients with renal failure. This is different from stem migration in non-renal failure patients as no osteolysis was seen on radiological images. Such migration might possibly be due to failure of the bone-cement interference and the osteoporotic bone in such patients that lacks adequate support [11]. Loosening of the stem is not related to the type of stem fixation (cemented or not cemented) and is also not related to the degree of renal failure. However, loosening might be linked to hyperparathyroidism in these patients [10,20]. Close follow-up after surgery is recommended to monitor renal function and cardiopulmonary complications to reduce the incidence of post-surgical complications [10].

Multiple reports in the literature that reported bilateral spontaneous fractures of the femoral neck in chronic kidney disease patients are summarized in Table 2.

**Table 2:** Summary of several published reports of bilateral femoral neck fractures in patients with chronic kidney disease. THA: total hip arthroplasty; CKD: chronic kidney disease

Author	Age	Serum creatinine	Serum urea	Serum vitamin D	Serum phosphate	Serum calcium	Serum parathyroid hormone	Treatment	Remarks
Zingraff et al., 1974 [21]	45	Data not available						Arthroplasty	Renal failure followed by a non-traumatic fracture
Gerster et al., 1983 [22]	69	2.6 mg/dL	No data available	22.8 ng/mL	1.2 mg/dL	8.1 mg/dL	220 pg/dL	Total hip replacement bilaterally with a Charnley prosthesis	Rheumatoid arthritis, and severe fluorosis was seen on histological examination
	78	1.6 mg/dL	No data available		3.4 mg/dL	8.9 mg/dL	420 pg/dL	Mode of fixation was not mentioned	
Öǧün et al., 2001 [23]	45	12.5 mg/dL	No data available			7.0 mg/dL	No data available	Open reduction and internal fixation with three cannulated hip screws	No non-union post-surgery, 4 years of follow-up
	35	5.5 mg/dL	No data available			7.5 mg/dL	No data available	Open reduction and internal fixation with three cannulated hip screws	The patient’s general condition deteriorated and was unable to comply with post-surgery rehabilitation physiotherapy. This led to stiffness but no failure of fixation
Hung et al., 2001 [24]	39	15.2 mg/dL	No data available		10.8 mg/dL	5.1 mg/dL	784 pg/dL	Bilateral staged hemiarthroplasty	Chronic hip pain followed by the diagnosis of renal failure. No major complications were noted over 3 years of follow up
Karapinar et al., 2003 [25]	23	5.65 mg/dL	107 mg/dL	8 ng/mL	6.7 mg/dL	7.9 mg/dL	147 pg/dL	Two-stage cemented THA	No remarks
Devkota et al., 2013 [26]	47	4.5 mg/dL	105.7 mg/dL	22 ng/mL	6.4 mg/dL	17.9 mg/dL	1,053.7 pg/dL	Non-surgical treatment	One year post-non-surgical treatment. No major complications. The patient was mobilized in a wheelchair
Garcia et al., 2014 [27]	43	No data available				7.1 mg/dL	>25 pg/dL	Simultaneous bilateral osteosynthesis	Tertiary hyperparathyroidism
Sathyana et al., 2015 [28]	23	8.9 mg/dL	175 mg/dL	No data available	6.2 mg/dL	4 mg/dL	488 pg/dL	Simultaneous bilateral uncemented modular bipolar hemiarthroplasty	The patient had osteoporosis and hypocalcemia-related seizures in the post-surgical period
Freitas et al., 2016 [29]	49	No data available				6.8 mg/dL	-	Simultaneous bilateral cementless THA	Secondary hyperparathyroidism
Mehmet et al., 2019 [30]	22	6.3 mg/dL	87 mg/dL	4 ng/mL	3.5 mg/dL	6.7 mg/dL	559 pg/dL	Bilateral THA	A bilateral fracture of the neck of the femur preceded the diagnosis of CKD
John et al., 2018 [15]	44	6.4 mg/dL	6.4 mg/dL	7.2 ng/mL	6.8 mg/dL	5.79 mg/dL	137 pg/dL	Two-stage bilateral cannulated screws fixation with washer	The patient expired three months after surgery due to renal and cardiac complications
	15	12.6 mg/dL	40 mg/dL	5.9 ng/mL	5.5 mg/dL	7.9 mg/dL	469 pg/dL	Two-stage procedure fixed with angle blade plate, valgus osteotomy, and fibula grafting	The postoperative course was uneventful
	64	5.0 mg/dL	148 mg/dL	16.34 ng/mL	6.6 mg/dL	7.1 mg/dL	252 pg/dL	Debridement and antibiotics due to the persistence of infection intraoperatively	The primary source of the infection was lung infection caused by Pseudomonas aeruginosa. The patient arrested during the dialysis session four weeks after discharge from orthopedic care
This study	37	3.57 mg/dL	13.3 mg/dL	57.2 ng/mL	2.56 mg/dL	2.18 mg/dL	157.7 pg/dL	Two-stage bilateral cementless total hip replacement	After two years of follow-up, the patient is doing well
Reference ranges	-	0.74–1.35 mg/dL	10–22 mg/dL	30–70 ng/mL	2.8–4.5 mg/dL	8.6–10.3 mg/dL	10–55 pg/dL		

## Conclusions

End-stage renal disease is a risk factor for complicated spontaneous neck of femur fractures that necessitate a multidisciplinary approach for treatment, along with preoperative optimization of patients’ general condition to reduce the risk of mortality and morbidity. The choice of implant should be customized based on each patient’s condition. Two-stage cementless THA is preferable in end-stage renal disease patients, especially in cases with associated hyperparathyroidism. Control of post-surgical comorbidities is crucial to avoid complications related to components such as loosening and further fractures, as well as systematic complications such as cardiopulmonary complications and sepsis related to implant infection.
